# 

**Published:** 1892-06-25

**Authors:** 


CONTENTS.

Is Vivisection Irreligious, Immoral, or Inhumane? - 193

Passing Topics.—A Centenary Collapse - 194

Medical History from the Earliest Times. By E. T. Withington, M.A., M.B.Oxon. XIV.—Heraclides and the Empiric School - 195

Everybody's Page - 196

The Practitioners' Mirror and Retrospect—

Medicine.—The Position of the Medical Examiner for Life Assurance.—II. - 202

Surgery.—Some of the Less Frequently Recognised Affections of Joints - 203

Practitioners' Bookshelf.—A Manual of Chemistry—Catechism Series—Anatomy - 204

New Drugs, Appliances, and Things Medical.—Wyeth's Beef Juice—The Anti-Medicine Taster—Bryonia - 204

Mr. Keir Hardie and the Miners - 197

Compulsory Vaccination - 198

Annotations.—The Health of Halifax—After Thoughts—Home Reading - 199

Notes and News - 200

Around the Hospitals—Scraps and Gleanings - 201

The Institutional Workshop—

Within the Hospitals. III.—The Royal Portsmouth, Portsea, and Gosport Hospital - 205

Hospital Opinion.—A Central Council - 206

"Suffering London."—Stirring addresses by Sir Andrew Clark and Mr. A. Egmont Hake - 206

The Hospitals Association - 208

The Student of Medicine.—Appointments—Vacancies—Pass Lists—Athletics

EXTRA SUPPLEMENT.—THE NURSING MIRROR.

En Passant. — Children—Holidays — The Q.V.J.I. in Dublin—Clapham Maternity Hospital—Private Nurses and their Ways—The Summer Number of the "Graphic"—Brabazon Home of Comfort—Wolverhampton Queen Victoria Nursing Institution—Poisons as Infantile Nourishment - lxxxvii

Ventilation, Disinfection, and Diet. By P. Caldwell Smith, M.D. XI.—Disinfection (*concluded*) - lxxxviii

Where to Go - lxxxviii

Nursing of the Sick English Soldier in India - lxxxix

Presentation - xc

Everybody's Opinion. — The Needless Dread of Nursing Small-pox Cases—Asylum Attendants—A Holiday in Switzerland - xc

Notes on Novelties - xc

Appointments - xc

Notes and Queries - xc

Nursing Homes. VII.—The Royal Portsmouth, Portsea, and Gosport Hospital - xci

For Reading to the Sick.—Coming - xci

Four Months in a Hospital Ward.—VI. - xcii

